# Exploring Risk Behaviors and Vulnerability for HIV among Men Who Have Sex with Men in Abidjan, Cote d′Ivoire: Poor Knowledge, Homophobia and Sexual Violence

**DOI:** 10.1371/journal.pone.0099591

**Published:** 2014-06-24

**Authors:** Josephine Aho, Avi Hakim, Bea Vuylsteke, Gisèle Semde, Honorat G. Gbais, Mamadou Diarrassouba, Marguerite Thiam, Marie Laga

**Affiliations:** 1 Department of Public Health, Institute of Tropical Medicine, Antwerp, Belgium; 2 Centers for Disease Control and Prevention, Atlanta, Georgia, United States of America; 3 Family Health International 360, Kinshasa, Democratic Republic of Congo; 4 Family Health International 360, Abidjan, Cote d′Ivoire; 5 Centers for Disease Control and Prevention, Abidjan, Cote d′Ivoire; 6 Program for Highly Vulnerable Populations, Ministry of Health and Fight against AIDS, Abidjan, Cote d′Ivoire; Rollins School of Public Health, United States of America

## Abstract

Men who have sex with men (MSM) are at high risk of HIV. Few data are available on MSM and HIV-related risk behaviors in West Africa. We aimed to describe risk behaviors and vulnerability among MSM in Abidjan, Cote d′Ivoire. We conducted a cross-sectional respondent-driven sampling survey with 601 MSM in 2011–2012. Sociodemographic and behavioural data as well as data related to emotional state and stigma were collected. Population estimates with 95% confidence intervals were produced. Survey weighted logistic regression was used to assess factors associated with inconsistent condom use in the prior 12 months. Most MSM were 24 years of age or younger (63.9%) and had attained at least primary education (84.4%). HIV risk behaviors such as low condom and water-based lubricant use, high numbers of male and female sex partners, and sex work were frequently reported as well as verbal, physical and sexual abuse. Inconsistent condom use during anal sex with a male partner in the prior 12 months was reported by 66.0% of the MSM and was positively associated with history of forced sex, alcohol consumption, having a regular partner and a casual partner, having bought sex, and self-perception of low HIV risk. MSM in Abidjan exhibit multiple and frequent HIV-related risk behaviors. To address those behaviours, a combination of individual but also structural interventions will be needed given the context of stigma, homophobia and violence.

## Introduction

Men who have sex with men (MSM) are known to be at high risk for HIV infection because of biologic and behavioural factors [1]–[3]. Studies have shown a much higher risk of HIV transmission among MSM (up to 18 times as high for unprotected receptive anal sex compared to unprotected vaginal sex) [4]–[6]. Consistent condom use with a water-based lubricant has been shown to decrease HIV transmission risk [7].

In sub-Saharan Africa, less data exist on MSM, likely due to the hidden nature of MSM in this region. Existing studies all show much higher HIV prevalence in MSM compared to the general population [1]. HIV-related risk behaviors such as multiple male and female sexual partnerships and frequent unprotected anal intercourse have been reported among MSM in African countries such as Namibia, Botswana, Malawi, Rwanda and Nigeria [8]–[10]. In addition, in predominantly hetero-normative sub-Saharan Africa, MSM generally suffer from high stigma, violence, and other human rights violations that can reinforce vulnerability, risky sexual behavior and thus HIV risk [7], [11].

The prevalence of HIV in Côte d′Ivoire is the second highest in West Africa, measured at 3.7% [12]. Sex between persons of the same sex is not illegal in Côte d′Ivoire. The Ministry of Health and the Fight against HIV/AIDS has defined MSM as a priority population in the National Strategic Plan for the Fight against HIV/AIDS 2011–2015. However, very few data exist on this population.

Abidjan, the economic capital of Cote d′Ivoire, has more than 6 million inhabitants, making it the biggest French-speaking West-African city, and the second biggest French-speaking city in the world. Despite the socio-political crisis that has shaken the country in the past two decades, Abidjan remains an important economic and sociocultural centre in Africa. As such, it has attracted numerous migrants from other regions of Cote d′Ivoire and from West Africa leading to high population diversity in terms of culture and ethnicity. The HIV prevalence in Abidjan is 5.1% for the adult population and 4.1% in the adult male population [13]. A facility-based study conducted in 2007–2008 in Abidjan on male sex workers who were mainly MSM reported a relatively high frequency of consistent condom use of more than 80% and a HIV prevalence of 50% [14].

The aim of this study is to describe risk behaviours for HIV related to number and type of sexual partners, condom and water-based lubricant accessibility and use, use of and engagement in sex work, and HIV vulnerability among MSM in Abidjan and to examine factors associated with unprotected anal intercourse in the preceding 12 months.

## Methods

### Ethics statement

All study participants gave written informed consent before starting study procedures. Data collected were anonymous. The study received approval from the National Research Ethics Committee of Côte d′Ivoire, from the Institutional Research Board of Family Health International 360, from the Ethics Committee of the University of Antwerp, Belgium, from the Institute of Tropical Medicine of Antwerp, Belgium, and from the US Centers for Disease Control and Prevention's Center for Global Health Associate Director for Science.

### Study design, population and settings

The study design included mixed methods. A bio-behavioural cross-sectional survey was conducted among MSM using respondent-driven sampling (RDS), and in-depth interviews were done among a subsample of men. The main focus was to determine HIV and sexually transmitted infections (STI) prevalence [15], as well as obtain a more in-depth understanding of sexual behaviour and other factors related to increased risk and vulnerability for HIV infection. This paper reports on the HIV-relevant risk behaviours and vulnerability.

MSM in Abidjan, the economical capital of Cote d′Ivoire, were invited to participate in this study. Being a MSM was defined as being a biological man who reported oral or anal sex with another man in the prior 12 months. Other inclusion criteria included age 18 years or older, being a resident of Abidjan for the preceding 6 months and speaking French. Sample size calculations were designed to detect a HIV prevalence of 20% with a precision of 10% and a type I error (alpha) of 0.05. The assumed study design effect was 2 [16]. The final required sample size was 600 participants.

### Recruitment and sampling

RDS is a chain referral sampling method that has been previously described and used for sampling of hard-to-reach populations [17]. Prior to the study, a formative assessment was conducted to assess feasibility and acceptability of the study methods for MSM. Study recruitment started with 5 participants chosen by the investigators (these first participants are called seeds in RDS terminology) who were socially well-connected with MSM and who were purposively selected for diversity in terms of age, education, occupation, income, area of residence, and prior HIV testing. Seeds were identified during the formative assessment and outreach leading up to the survey. Six more seeds were added later to speed up recruitment and in an attempt to reach more hidden sub-groups of the population, including older MSM. Each seed received three coupons and was invited to recruit three eligible candidate participants who, in turn, received three recruitment coupons and so on, until the sample size was attained. Each pre-printed coupon with unique traceable numbers could be linked to the recruiter and the recruit using RDS coupon manager software (RDSCM 3.0, CDC, Atlanta, Georgia). The recruitment took place from October 2011 to February 2012. Two static sites located in the north and south of Abidjan (one NGO-operated health facility and the office of an NGO working in HIV prevention and with HIV-infected individuals) were chosen during formative assessment as study sites. Rooms were rented in these facilities to conduct study procedures separately from the facilities' daily activities. Candidate participants could enrol at either site. The same staff alternated between sites to reduce double enrolment and ensure consistency of methods.

Candidate participants with a valid coupon were screened for eligibility upon arrival at the study site. Only participants who completed the main interview received the 3 coupons. Those participating near the end of the recruitment period received no coupons as the required sample size was almost attained. Participants received 3,000 francs CFA (6 US dollars) for transport and 2,000 francs CFA (4 US dollars) for time spent for the first visit as a participation incentive. Participants in the qualitative data collection received the same amounts. At the second visit, participants in the RDS survey received 1,000 francs CFA (2 US dollars) per eligible participant recruited (up to a maximum of 3). All participants received free condoms, water-based lubricants and written HIV prevention material.

### Data collection

Data were collected by a trained interviewer using a structured close-ended questionnaire. Questionnaire Design Studio software 2.6.1 (Nova, Bethesda, Maryland, USA) was used to conduct computer-assisted personal interviews. Interview questions included sociodemographic characteristics, sexual behavior, exposure to HIV services, and self-perceived stigma related to being an MSM. Data were also collected on participants' social network size using the following question “how many MSM 18 years or older living in Abidjan, who you know by name or nickname and who know you by your name or nickname, have you seen in the past 30 days?”. The personal network sizes derived from the final question and recruitment probabilities were used for weighting and adjustment of RDS data to allow for computation of estimated population proportions.

A mixed methods design in which qualitative and quantitative data collection occurred simultaneously and where qualitative data provide an explanatory support for quantitative data [18] was used to assess risk profiles and patterns of vulnerability. Qualitative data were collected through individual semi-structured interviews on a subset of 30 purposively selected participants in the RDS study for maximum variation. The participants were selected on the basis of their answers to the quantitative data collection and to complement previously collected qualitative data. The selection was based on sociodemographics (for e.g., being an older MSM, being married to a woman), risk behaviors (having high number of sexual partners, using drug, selling sex, always or never using condoms), history of abuse and emotional state (having been sexually abused, having been stigmatized by a health agent, by a police officer or by other MSM, feeling depressed) and access to prevention and treatment (having never been tested for HIV, being HIV-infected, having had multiple sexually transmitted infections). Data on self-perception, gender-based violence and stigma and sexual behavior were collected. Interviews were conducted in French, recorded, and transcribed in Word format.

### Data analysis

Sample proportions were calculated with SPSS 17.0 (SPSS Inc., Chicago, US). Estimated population proportions and 95% confidence intervals (CI) were calculated using Respondent-Driven Sampling Analysis Tool (RDSAT 6.0.1; www.respondentdrivensampling.org; Ithaca, US) with the dual component estimator [17], [19], enhanced data smoothing, and 15,000 iterations. Equilibrium (the fact that the sample composition does not change with the inclusion of new recruits [17]) was attained for all main variables of interest with a convergence radius of 0.02. Sociodemographic characteristics are described along with sexual behavior i.e., type of sexual behavior (oral or anal sex), number, type (regular, casual or commercial) and sex of sexual partners, and condom and water-based lubricant use and access to these prevention tools. Stigma and emotional state as factors of HIV vulnerability are also described. Data from seeds were included in all analyses.

We also assessed variables associated with unprotected anal intercourse, our main outcome of interest, using analytic survey methods. Unprotected anal intercourse was defined as not having consistently used a condom for receptive or insertive anal sex with any regular or casual male partner in the past 12 months. Missing values were rare and did not exceed 6% for any variable. They were assumed to be missing at random and excluded from the analysis. Logistic regression was performed using individualized weights for the dependent variable exported from RDSAT 6.0.1 for a survey binary logistic regression in STATA 12.1 (StataCorp, College Station, TX, USA). The following variables were considered as potential correlates of unprotected anal intercourse: behavior (alcohol consumption in the past 30 days and frequency of alcohol consumption before sex with the last male partner, and drug use in the past 12 months, number and type of sex partners, role (insertive or receptive) during anal sex with the last partner, having sold or bought sex), emotional state measured by PHQ-2 screen for depression with a cut point of 3 (Patient Health Questionnaire-2 (PHQ-2) is a first-step two-items screener for depression that inquires about depressed mood and loss of interest or pleasure in routine activities over the past 2 weeks) [20], , history of abuse (history of coerced sex or physical abuse related to the MSM status according to the participant), exposure to preventive interventions (HIV test in the preceding 12 months, result of the last HIV test result received, contact with a health educator in the preceding 12 months and knowledge of HIV), reported STI symptoms in the past 12 months and self-perceived HIV risk. A score was calculated to measure knowledge of HIV by computing the number of correct answers to 4 questions on HIV risk and treatment. Thus, the maximum knowledge score was 4 correct answers. Sociodemographic characteristics (age, marital status, monthly income, education, occupation, sexual orientation) were also included in the analysis as potential confounders. Results are presented as odds ratios (OR) with 95% CI and *P* value. Variables associated with the dependent variable in the survey bivariate analysis with a *P*<0.1 were included in a survey multivariate logistic regression. Multivariate logistic regression was performed on individuals with complete data for variables included. People who self-reported being HIV-infected during the questionnaire (19 participants) were excluded from multivariate analysis to allow inclusion of self-perceived risk of HIV infection in the multivariate model. Goodness of fit was assessed using Hosmer and Lemeshow test and the model's chi square. Adjusted OR and their 95% CI are presented. A *P*<0.05 was considered statistically significant.

Qualitative data were analyzed as explanatory support for quantitative findings using content analysis. Throughout data collection, qualitative data were reviewed and two raters identified concepts and themes to develop a codebook used by 4 raters to code interviews. Each interview was coded by two different raters using Dedoose software (SCRC, Los Angeles, United States). Recurrent themes were identified and presented with illustrative quotes.

## Results

### Recruitment

The study enrolled 11 seeds, 2 of which were unproductive, meaning that they were unable to recruit any eligible participants. During the 15 weeks of recruitment, 1608 coupons were issued and a total of 643 persons, including the 11 seeds, were screened. Among those 643 candidate participants, 603 (93.8%) were eligible. The main reason for ineligibility was not ever having had sex with a man (11/40) or having had last sex with a man more than 12 months before recruitment (11/40). The maximum number of waves for a recruitment chain (maximum length of a recruitment chain i.e. maximum number of successive generations of recruits) was 12. Of the 603 eligible, 601 participants completed the behavioral questionnaire and were included in the analysis. The estimated design effect for unprotected intercourse with men in the past 12 months was 2.33 [16].

### Characteristics of the population, emotional state and history of abuse

Sociodemographic characteristics, sexual identity, emotional state and history of abuse are presented in Table 1. In weighted analysis, a majority of MSM was between 18 and 24 years old (63.9%; 95% CI: 57.2–70.5). Education level was high with 59.6% (95% CI: 54.3–66.7) having been to secondary school and one-quarter having accessed post-secondary education (24.8%; 95% CI: 19.1–29.7).

**Table 1:** Sociodemographic characteristics, emotional state, history of abuse and other descriptives of MSM in Abidjan (N = 601).

Variables	Unweighted^a^ % (n)	Weighted^b^ % (95% CI)
**Sociodemographic characteristics and self-reported STD symptoms**		
Age (median 23, range 18–51)		
18–24	59.1 (355)	63.9 (57.2–70.5)
25–29	25.8 (155)	23.8 (18.5–29.4)
30–34	9.1 (55)	8.7 (5.1–12.1)
35–39	3.8 (23)	2.8 (1.2–5.5)
40+	2.2 (13)	0.8 (0.1–1.7)
Nationality		
Ivoirian	95.0 (571)	95.0 (92.9–97.2)
Other	5.0 (30)	5.0 (2.8–7.2)
Marital status		
Never married	91.5 (550)	92.4 (89.5–95.3)
Married	0.8 (5)	0.9 (0.0–2.3)
Divorced, separated, widow	0.3 (2)	0.2 (0.0–0.7)
Married or cohabitating with a woman	2.7 (16)	2.9 (1.2–4.7)
Cohabitating with a man	4.7 (28)	3.6 (1.7–5.7)
Highest education level started		
Never been to school	5.8 (35)	6.3 (3.5–8.8)
Primary	7.0 (42)	9.3 (5.8–13.1)
Secondary	55.9 (336)	59.6 (54.3–66.7)
Post-secondary	31.3 (188)	24.8 (19.1–29.7)
Work status		
Unemployed	14.5 (87)	17.5 (12.5–22.2)
Student	40.4 (243)	40.6 (34.7–46.6)
Shopkeeper, retailer, hotel worker	18.6 (112)	14.5 (10.8–19.2)
Laborer, driver, artist	15.0 (90)	17.2 (12.4–22.7)
Clerical, professional	5.1 (31)	4.6 (2.4–6.9)
Sex workers	0.7 (4)	0.1 (0.0–0.3)
Other	5.7 (34)	5.5 (3.2–8.2)
Sexual identity		
Homosexual	43.9 (264)	40.2 (34.7–46.4)
Bisexual	54.4 (327)	57.6 (51.4–62.8)
Heterosexual	1.4 (8)	1.8 (0.5–3.9)
Don't know	0.3 (2)	0.4 (0.0–1.5)
Age at first sex with a man		
≤10	5.6 (31)	6.0 (2.9–9.4)
11–15	21.7 (121)	18.6 (14.4–24.2)
16–20	54.2 (302)	50.2 (43.2–56.8)
21–25	12.9 (72)	17.4 (11.8–22.8)
26–30	5.0 (28)	6.8 (3.5–11.0)
>31	0.6 (3)	1.1 (0.0–2.9)
Self-reported STD symptoms in the past 12 months	19.0 (114)	19.2 (14.5–24.0)
**Emotional state and history of abuse**		
Dominant feeling related to whole life in general		
Positive feeling	46.0 (276)	44.1 (38.7–50.8)
Ambivalent feeling	13.8 (83)	11.5 (8.3–14.7)
Negative feeling	40.2 (241)	44.3 (38.0–50.1)
PHQ-2 Screen for depression in the past 2 weeks		
Screen not depressed	74.7 (447)	74.3 (69.9–78.8)
Screen depressed	25.3 (151)	25.7 (21.2–30.1)
History of harassment or abuse because of MSM status	44.9 (270)	38.5 (32.4–43.6)
History of verbal harassment (threats, insults) because of MSM status	40.9 (246)	33.0 (27.5–37.9)
History of emotional abuse (isolation, exclusion) because of MSM status	9.5 (57)	6.3 (3.9–8.9)
History of physical abuse because of MSM status	13.8 (83)	8.5 (5.5–11.4)
History of forced sex	24.5 (147)	21.4 (16.6–26.0)
**Alcohol and drug use**		
Alcohol use in the past 30 days		
Never	27.4 (165)	32.4 (26.0–38.3)
Once a week or less	49.1 (295)	18.4 (14.5–22.9)
More than once a week	23.1 (139)	49.1 (43.4–55.6)
Non-intravenous drug use in the past 12 months	9.3 (56)	9.6 (6.2–13.5)
**Exposure to HIV interventions**		
HIV test in the past 12 months	37.9 (228)	32.1 (26.6–37.0)
Had had an education session with a NGO or a health agent in the past 12 months (not related to HIV testing)	48.1 (289)	39.9 (34.4–45.5)

a. Unweighted: sample proportions.

b. Weighted: population proportions.

Almost all MSM self-identified as bisexual (57.6; 95% CI: 51.4–62.8) or homosexual (40.2, 95% CI: 34.7–46.4). A large proportion (38.5%; 95% CI: 32.4–43.6) had experienced harassment or abuse. Threats and insults related to MSM status in particular were common (33.0%; 95% CI: 27.5–37.9). Forced sex was also frequent (21.4%; 95% CI: 16.6–26.0). Many MSM had negative feelings about their life as a whole (44.3%; 95% CI: 38.0–50.1) and 25.7% (95% CI: 21.2–30.1) screened as depressed for the preceding 2 weeks according to the PHQ-2. All the 30 qualitative interview participants reported experiencing violence, stigma or discrimination from family, friends, co-workers or strangers because of their MSM status, or knowing a MSM who had: “*Our close neighbors, they're always bad-mouthing, slandering. They generally say that gays are damned, they are losers, aliens. Being gay is synonym of all that is negative, of all that is lost in this world. He is a synonym of malediction and horror*” Unemployed man, 32 years old. Many participants felt they had no legal recourse in cases of violence or discrimination: “*When we go to lodge a complaint at the police station, it doesn't go anywhere. They don't care. They say:” they are gay, they are not worth it”* ” Unemployed man, 32 years old. MSM try to hide their sexual identity or behavior or find themselves in conflicts with their entourage if they choose to unveil it: “*People were talking in the neighborhood. (…) My mom asked me again and again. In the end, I said: yes, I am gay. It went really really badly. What she said… that is one thing… but what she did… I got the impression I was in front of someone who found me in the street… someone who didn't know me… (Silence). Those are really difficult things*” Student, 21 years old. Many MSM reported stress, depression and frustration.

### Knowledge and self-perceived HIV risk

Overall HIV knowledge was low (Table 2). The increased risks associated with anal sex were known by 38.8% (95% CI: 32.6–44.0) of the MSM. Most MSM perceived themselves at low risk of HIV infection (59.1%; 95% CI: 53.0–64.5) (Table 2).

**Table 2:** Knowledge, HIV risk perception and reported sexual behavior of MSM in Abidjan (N = 601)^a^.

Variables	Unweighted % (n)	Weighted % (95% CI)
**Knowledge of HIV**		
Risk of HIV transmission during sex with a man compared to sex with a woman		
More risky with women	18,6 (112)	22,9 (17.7–27.9)
More risky with men	14,5 (87)	12,8 (9.3–16.1)
Same risk for both	66,1 (397)	63,3 (58.0–69.1)
Do not know	0,8 (5)	1,1 (0.1–2.5)
Type of sex that puts one at the most risk for HIV		
Manual	0.5 (3)	0.8 (0.0–2.2)
Oral	8.3 (50)	8.2 (5.2–11.5)
Vaginal	46.1 (277)	50.6 (45.3–57.2)
Anal	43.3 (260)	38.8 (32.6–44.0)
Between the thighs	1.0 (6)	0.4 (0.1–1.0)
Do not know	0.8 (5)	1.2 (0.1–2.7)
Most risky type of anal sex		
Insertive (active) anal sex	5.7 (34)	6.9 (3.9–10.1)
Receptive (passive) anal sex	37.9 (228)	38.5 (32.8–44.2)
Both active and passive anal sex have the same risk	55.6 (334)	53.3 (48.0–59.1)
Both active and passive anal sex have no risk	0.5 (3)	0.6 (0.0–1.4)
Knows there is treatment for HIV	94.0 (565)	91.3 (87.2–94.7)
Score of HIV knowledge		
0 correct answer	2.2 (13)	2.3 (1.0–3.9)
1 correct answer	32.4 (195)	35.2 (30.0–41.5)
2 correct answers	44.9 (270)	45.4 (39.2–50.7)
3 correct answers	14.5 (87)	11.8 (8.5–15.3)
4 correct answers	6.0 (36)	5.2 (3.2–7.7)
**Self-perceived risk of HIV infection**		
Knows an HIV positive MSM	20.0 (120)	9.9 (7.2–13.0)
Self-perceived HIV risk		
No risk	18.3 (110)	19.6 (15.2–25.3)
Low risk	58.1 (349)	59.1 (53.0–64.5)
High risk	19.5 (117)	17.7 (13.4–22.3)
Already HIV+	3.1 (19)	2.4 (0.9–4.4)
Don't know	1.0 (6)	1.1 (0.1–2.6)
**Sexual behavior**		
**a) With men in the past 12 months**		
Type of sex		
Oral sex	2.7 (16)	2.9 (1.2–4.5)
Anal sex	6.3 (38)	6.8 (4.4–9.3)
Oral and anal sex	91.0 (547)	90.3 (87.7–93.3)
Number of male anal sex partners (n = 585) (median 4, range 1–100)		
1	22.6 (132)	27.7 (22.9–33.3)
2–3	32.7 (191)	37.4 (31.8–42.9)
4–5	17.1 (100)	15.7 (12.1–19.7)
6–9	11.5 (67)	9.7 (6.3–13.5)
10+	16.1 (94)	9.5 (6.7–12.4)
Number of regular male anal sex partners (n = 585)		
0	18.3 (107)	21.8 (16.8–27.7)
1	46.5 (272)	47.2 (40.5–52.7)
2	18.1 (106)	19.9 (15.7–25.8)
3+	16.9 (99)	11.1 (7.6–14.6)
Number of casual male anal sex partners (n = 585)		
0	30.3 (177)	32.2 (27.2–38.0)
1	24.1 (141)	27.8 (22.2–33.2)
2	14.9 (87)	13.6 (9.6–18.0)
3+	30.7 (179)	26.4 (21.2–31.6)
Frequency of condom use (n = 585)		
Always	32.6 (191)	34.0 (28.4–39.8)
Sometimes	59.5 (348)	56.6 (50.8–62.6)
Never	7.9 (46)	9.4 (5.9–13.1)
Reasons for never using a condom (n = 46)		
To avoid diminishing sexual pleasure	34.8 (16)	31.9 (12.8–53.5)
Out of trust of their partners	32.6 (15)	27.0 (13.5–48.4)
Ashamed of buying condoms	8.7 (4)	12.4 (0.8–28.3)
Latex allergy	6.5 (3)	5.2 (0.0–13.6)
Consistent water-based lubricant use (n = 601)	22.8 (137)	20.5 (16.4–24.9)
Consistent condom and water-based lubricant use (n = 585)	10.2 (60)	9.9 (6.9–13.7)
Non water-based lubricants used^b^		
Saliva	56.2 (298)	56.5 (49.3–62.4)
Vaseline	37.5 (199)	37.7 (32.1–44.9)
Shea butter	38.5 (204)	39.6 (34.4–46.9)
Hand or body lotion	37.9 (201)	33.5 (27.4–39.6)
Butter, cooking oil	14.9 (79)	14.4 (10.5–18.4)
History of condom breakage (n = 539)	47.7 (257)	41.7 (35.7–47.3)
**b) With the last male partner**		
Type of partner		
Regular	59.7 (359)	59.6 (54.3–66.0)
Casual	32.6 (196)	32.5 (26.6–37.5)
Participant paid the partner for sex	1.2 (7)	1.0 (0.2–2.4)
Participant was paid for sex	6.5 (39)	6.9 (4.0–9.7)
Alcohol consumption before sex		
Always	7.7 (46)	9.5 (6.2–13.9)
Sometimes	41.6 (250)	38.5 (32.4–44.2)
Never	50.2 (302)	52.0 (45.7–58.1)
Condom use at last sex (n = 585)	64.8 (379)	63.2 (57.4–69.3)
Reasons for not using a condom at last sex (n = 497)		
Did not have a condom at time of intercourse	38.0 (189)	35.0 (29.1–41.5)
Partner was against using condoms	9.9 (49)	10.3 (6.6–14.8)
Did not feel at risk because had only one partner	8.0 (40)	7.1 (4.1–11.0)
Did not think about using a condom	7.2 (36)	6.7 (4.0–9.7)
Did not feel at risk for other reasons	8.4 (42)	6.7 (4.2–9.6)
Do not like condoms	3.4 (17)	5.5 (2.4–9.3)
Condoms are too expensive	1.2 (6)	0.6 (0.1–1.2)
Role during anal sex (n = 585)		
Active or insertive	39.1 (235)	42.8 (37.0–49.9)
Passive or receptive	47.4 (285)	45.1 (38.1–51.3)
Both	10.8 (65)	12.1 (8.3–15.9)
**c) With women in the last 12 months**		
Number of regular female sex partners for vaginal or anal sex		
0	61.6 (370)	56.0 (49.6–62.1)
1	24.6 (148)	31.0 (25.1–37.4)
2	9.0 (54)	8.2 (5.8–11.6)
3+	4.8 (29)	4.7 (2.6–6.8)
Number of casual female sex partners for vaginal or anal sex		
0	66.9 (402)	63.2 (57.1–68.6)
1	14.8 (89)	17.3 (12.8–21.2)
2	7.3 (44)	7.7 (4.9–11.3)
3+	11.0 (66)	11.8 (8.5–16.7)
Condom use at last sex with a woman (n = 304)	62.5 (190)	60.3 (52.0–68.1)
d) **Sex work**		
Have given money or goods in exchange of sex with a man in the past 12 months	11.0 (66)	8.3 (5.5–10.7)
Have sold sex in exchange of money or goods in the past 12 months	29.3 (176)	23.4 (19.5–27.2)

a. Numbers do not add up to 100% because of missing data or rounding.

b. More than one answer possible.

### Sexual behavior with men

Almost all MSM (90.3%; 95% CI: 87.7–93.3) had anal and oral sex in the preceding 12 months, and 2.9% (95% CI: 1.2–4.5) had only oral sex. Almost one-tenth of MSM (8.3%; 95% CI 5.5–10.7) bought sex and 23.4% (95% CI: 19.5–27.2) sold sex in the last 12 months.

Multiple sexual partnerships were common, with a third of MSM (34.9%; 95% CI: 32.6–36.9) having had more than 3 male anal sex partners in the year preceding the study. Although the total number of casual male partners was higher than the total number of regular male partners, more MSM had regular partners than casual partners. According to most qualitative survey participants, frequent one-time sex or group-sex explained the high numbers of male and female casual sex partners.

One-third of MSM (34.8%; 95% CI: 29.3–41.0) consistently used condoms during anal sex in the last 12 months, while only 9.9% consistently used a condom with a water-based lubricant (95% CI: 6.9–13.7). Among those who used condoms at least once, 41.7% (95% CI: 35.7–47.3) reported a condom breakage in the preceding 12 months. Those who never used a condom in the last 12 months (9.4%; 95% CI: 5.9–13.1) did so mainly to avoid diminishing sexual pleasure (31.9%; 95% CI: 12.8–53.5) or out of trust of their partners (27.0%; 95% CI: 13.5–48.4).

### Sexual behavior with women

Sex with a woman was common among MSM with 56.1% (95% CI: 53.6–58.9) reporting sex with a woman in the preceding 12 months. Over one-quarter of MSM (29.0%; 95% CI: 22.3–37.2) had at least 3 female partners in the preceding year. According to qualitative interviews, some MSM have sex with women because they are sexually attracted to them but for others, sex or relationships with women are used to hide their MSM status: *“At a certain age, some guys become bi* (bisexual)… *They are gay when they are young and then… I don't know if it is because of the responsibilities or what (…) but after 30, 35 years old, they get married, have kids. but still they go on. I think it is mostly their jobs that ask for that… I can't really understand… or it can be family pressure”* Cook, 42 years old.

A condom was used at last sex with a woman by 60.3% of the MSM (95% CI: 52.0–68.1).

### Access to condoms and water-based lubricants

Not having a condom available before or during intercourse was the main reason for not using a condom at last sex with a man (35.0%; 95% CI: 29.1–41.5) (Table 2). Also, 43.4% (95% CI: 37.7–49.4) of MSM were unable to obtain a condom when needed at least once in the preceding 12 months mainly because the convenience store was closed (38.7%; 95% CI: 30.9–47.4) or too far away (32.4; 95% CI: 24.5–41.4). The main reason (21.2%; 95% CI: 14.8–27.2) for not using water-based lubricants was their perceived high cost. Qualitative data also showed that some respondents are ashamed of purchasing water-based lubricants as it may be associated to being gay by the sellers.

### Factors associated with unprotected anal intercourse

We investigated factors associated with unprotected receptive or insertive anal intercourse with male partners in the prior 12 months (Table 3). In bivariate analysis, the following variables were positively associated with unprotected anal intercourse at p<0.05: history of physical abuse because of MSM status, history of forced sex, alcohol consumption in the prior 30 days, self-reported STD symptoms in the past 12 months, having had one or more casual male partners in the past 12 months, having bought sex from a man in the last 12 months, and low or high self-perception of HIV risk. The following factors remained significantly associated with increased odds of inconsistent condom use at p<0.05 in multivariate analysis: history of forced sex (OR: 2.64; 95% CI: 1.23-5.65), having consumed alcohol in the past 30 days (OR: 2.05; 95% CI: 1.14–3.69), having had a regular male partner in the past 12 months (OR: 1.93; 95% CI: 1.01–3.66), having had one (OR: 2.04; 95% CI: 1.03–4.01) or more (OR: 2.61; 95% CI: 1.22–5.60) casual male partners in the past 12 months, having bought sex from a man in the past 12 months (OR: 6.15; 95% CI: 1.92-19.74) and low (OR: 2.14; 95% CI: 1.11–4.11) or high risk perception (OR: 6.00; 95% CI: 2.31–15.63) of HIV infection.

**Table 3:** RDS weighted associations between unprotected anal intercourse with a male partner in the past 12 months and selected variables.

Variables	% inconsistent condom use	OR (95% CI)	P value for bivariate OR	Adjusted OR (95% CI) (N = 560)	P value for multivariate OR
Age					
18–24	65.7	Ref.	0.888	-	-
25+	64.9	0.96 (0.59–1.59)			
Nationality					
Ivoirian	65.3	Ref.	0.604	-	-
Other	70.4	1.30 (0.48–3.46)			
Marital status					
Never married, divorced, separated, widow	65.3	Ref.	0.417	-	-
Married or cohabitating with a woman	56.5	0.67 (0.21–2.16)			
Cohabitating with a man	81.0	2.09 (0.57–7.72)			
Highest education level started					
Never been to school/Primary	73.0	Ref.	0.280	-	-
Secondary/Post-secondary	64.2	0.67 (0.32–1.39)			
Work status					
Unemployed	65.3	Ref.	0.531	-	-
Student	62.0	0.85 (0.40–1.81)			
Employed	68.7	1.15 (0.53–2.48)			
Sexual identity					
Homosexual	69.1	Ref.	0.480	-	-
Bisexual	62.7	0.74 (0.45–1.23)			
Heterosexual	60.0	0.57 (0.07–4.27)			
History of verbal harassment (threats, insults) because of MSM status					
No	62.1	Ref	0.083	Ref	
Yes	72.3	1.59 (0.94–2.68)		1.67 (0.89–3.16)	0.108
History of physical abuse because of MSM status					
No	63.7	Ref.	0.011	Ref.	0.532
Yes	84.0	2.91 (1.28–6.63)		1.39 (0.49–3.94)	
History of forced sex					
No	60.5	Ref.	< 0.001	Ref.	
Yes	83.3	3.33 (1.75–6.32)		2.64 (1.23–5.65) *	0.012
Dominant feeling related to whole life in general					
Positive feeling	59.8	Ref	0.059	Ref	
Ambivalent feeling	78.1	2.36 (1.13–4.94)		1.39 (0.59–3.30)	0.448
Negative feeling	68.1	1.45 (0.86–2.43)		1.23 (0.71–2.16)	0.460
Alcohol use in the past 30 days					
Never	52.2	Ref.	0.007	Ref.	
Once a week or less	70.7	2.23 (1.26–3.94)		2.05 (1.14–3.69)	0.017
More than once a week	74.4	2.66 (1.32–5.35)		2.48 (1.13–5.44)	0.023
Non-intravenous drug use in the past 12 months					
No	63.9	Ref.	0.113	-	-
Yes	78.9	2.11 (0.84–5.30)			
Self-reported STD symptoms in the past 12 months					
No	62.7	Ref.	0.046	Ref.	
Yes	76.5	1.94 (1.01–3.74)		1.05 (0.46–2.37)	0.906
HIV test in the past 12 months					
No	66.3	Ref.	0.666	-	-
Yes	63.8	0.90 (0.55–1.47)			
Last HIV test result received					
Negative	69.3	Ref.		-	-
Positive	66.7	0.51 (0.20–1.30)	0.166		
Had had an education session with a					
NGO or a health agent in the past 12 months (not related to HIV testing)					
No	62.4	Ref.	0.145	-	-
Yes	70.2	1.43 (0.88–2.30)			
Score of HIV knowledge					
0 correct answer	46.3	Ref.	0.264	-	-
1 correct answer	72.8	3.11 (0.03–11.72)			
2 correct answers	62.6	1.94 (0.52–7.21)			
3 correct answers	60.8	1.80 (0.44–7.29)			
4 correct answers	58.9	1.67 (0.35–8.00)			
Knows an HIV positive MSM					
No	57.0	Ref.	0.126	-	-
Yes	60.3	1.09 (0.98–1.22)			
Had at least one regular male anal sex partner in the past 12 months					
No	54.8	Ref.	0.059	Ref.	
Yes	68.4	1.79 (0.98–3.28)		1.93 (1.01–3.66) *	0.045
Number of casual male anal sex partners in the past 12 months					
0	52.7	Ref.	0.003	Ref.	
1	66.3	1.76 (0.93–3.34)		2.04 (1.03–4.01) *	0.040
2	68.8	1.98 (0.92–4.25)		1.83 (0.81–4.13)	0.145
3+	78.7	3.28 (1.73–6.21)		2.61 (1.22–5.60) *	0.014
Role during anal sex with last partner					
Active or insertive	60.0	Ref.	0.247	-	-
Passive or receptive	69.6	1.54 (0.91–2.60)			
Both	68.6	1.45 (0.67–3.15)			
Had at least one regular female partner for vaginal or anal sex in the past 12 months					
No	66.8	Ref.	0.571	-	-
Yes	63.4	0.87 (0.53–1.42)			
Have sold sex in exchange of money or goods in the past 12 months					
No	76.0	Ref.	0.177	-	-
Yes	72.1	1.47 (0.84–2.56)			
Have given money or goods in exchange of sex with a man in the past 12 months					
No	63.1	Ref.	0.004	Ref.	
Yes	91.8	6.63 (1.85–23.74)		6.15 (1.92–19.74) *	0.002
Use of free condoms					
Yes	62.7	Ref.	0.608	-	-
No	61.6	0.99 (0.53–1.85)			
Never received free condoms	65.2	1.43 (0.69–2.95)			
Self-perceived HIV risk					
No risk	40.9	Ref.	<0.001	Ref.	
Low risk	67.8	3.02 (1.65–5.55)		2.14 (1.11–4.11) *	0.023
High risk	85.4	8.50 (3.72–19.39)		6.00 (2.31–15.63) *	<0.001
Don't know	28.6	0.58 (0.06–5.96)		-	

* p<0.05.

## Discussion

To our knowledge, this RDS-based study is the first to document population-level estimates of HIV-related risk behaviors and vulnerability among MSM in Côte d′Ivoire and one of the first in West Africa.

Our findings show a high prevalence of HIV-related risk behaviors among MSM in Abidjan, including unprotected anal intercourse and inconsistent water-based lubricant use, multiple sexual male and female partnerships, and commercial sex. Such high levels of risk behaviors among MSM have been reported in other African populations [2], [8]–[10]. Our findings are supported by the high HIV prevalence of 18% reported in this population [15]. Highly frequent risk behaviors in the context of high HIV prevalence in this population merits public health attention and intervention. Moreover, the fact that more than half of MSM had a female partner makes this population a potential “bridge” population to other population sub-groups at lower risk of HIV infection.

Despite these high-risk behaviors, most MSM perceived themselves at low risk of HIV infection. In another study, having poor knowledge of MSM-specific HIV information was associated with higher odds of inconsistent condom use [22], but poor knowledge was not associated with inconsistent condom use in our study. Having had at least one education session with a peer educator did not support consistent condom use, as one session may not be enough to have an impact.

These results will be useful for planning prevention and other services targeting MSM in Abidjan and in other cities of West Africa. In particular, messages addressing the importance of using condoms and water-based lubricants and HIV risks associated with anal sex in this population should be provided. Considering the reporting of frequent condom breakage, condom-compatible lubricants should be made more accessible geographically and financially and its use promoted as its purchase is viewed as stigmatizing.

In our study, high-risk behaviors are undertaken in a context of verbal, physical and sexual violence, and expression of negative feelings on life and depression. History of forced sex was independently associated with higher odds of unprotected anal intercourse in this study. These various types of violence and stigma may increase MSM vulnerability and difficulty to control their own risk of acquiring HIV [23]–[25]. Studies in North America and sub-Saharan Africa have shown that MSM reporting social discrimination due to their sexual behavior or orientation are more likely to engage in high-risk sex [11], [26]. This study highlights the need for structural interventions addressing frequent human rights violations that can increase risk-taking behaviors and hinder access to prevention and care for MSM in Cote d′Ivoire. Stigma and violence associated with same-sex behavior can lead to internalized homophobia defined as self-hatred and shame [27] which in turn may prevent people from accessing prevention services and lower their agency and motivation to adopt safer sex behaviors [28], [29]. Low self-esteem could also explain the association between risk perception and unprotected anal intercourse found in our study.

This study has some limitations. First, with RDS, biases can occur if recruitment does not penetrate all sub-groups. This may be the case in our study as older MSM were reported in qualitative interviews to be less likely to participate than others out of fear of disclosure of their MSM status. Higher participation of younger MSM in similar studies has been reported in sub-Saharan Africa [8], [30]. Population estimates of this study should thus be interpreted with caution for older persons in the MSM population.

Second, the study is based on behaviors reported in a face-to-face interview and may be prone to social desirability responses. This has been minimized through comprehensive training of study staff to help participants feel at ease answering questions. Nevertheless, individuals may tend to under-report risk behaviors. We thus believe that our estimates may be conservative.

Third, with a cross-sectional survey, temporal ordering and thus causality of associations cannot be assessed. For example, forced sex may have occurred after a year of inconsistent condom use and thus, may not have contributed to condoms use or non-use.

In conclusion, this study is one of the first of this size in sub-Saharan Africa, thanks in part to the involvement and commitment of MSM and NGOs serving MSMs in Abidjan to participating in the study and having more data on their population. The results of this study show high HIV risk and vulnerability among MSM in Abidjan and highlight the urgent need for more MSM-tailored and targeted prevention interventions. It is now clear that MSM are an important additional key population for HIV prevention and treatment in sub-Saharan Africa and that individual and societal approaches will be needed to address further spread of HIV.
